# Lectures on the Theory and Practice of Physic

**Published:** 1849-01

**Authors:** 


### RTICLE IV.

*Lectures on the Theory and Practice of Physic.* By JNO. BELL, M. D., Member of the American Medical Association, and of the Med. Society of the State of Pennsylvania, Fellow of the College of Physicians of Philadelphia, &c., &c.; and by WILLIAM STOKES, M. D., Lecturer at the Medical School, Park Street, Dublin, Physician to the Meath Hospital, &c., &c. Fourth edition revised and enlarged, in two volumes, pp. ; Philadelphia: Ed. Barrington & Geo. D. Haswell, 1848. (From the publishers, and for sale by J. Keene & Bro., Chicago.)

The fact of there being a demand for a fourth edition of Stokes' and Bell's Practice is sufficient evidence that the work is highly favored by practitioners and students as a book of reference and text book.

It contains, doubtless, as much if not a greater amount of useful and correct information than any other systematic work of the kind. H.

